# Using Molecular Data for Epidemiological Inference: Assessing the Prevalence of *Trypanosoma brucei rhodesiense* in Tsetse in Serengeti, Tanzania

**DOI:** 10.1371/journal.pntd.0001501

**Published:** 2012-01-31

**Authors:** Harriet K. Auty, Kim Picozzi, Imna Malele, Steve J. Torr, Sarah Cleaveland, Sue Welburn

**Affiliations:** 1 Division of Pathway Medicine and Centre for Infectious Diseases, School of Biomedical Sciences, College of Medicine and Veterinary Medicine, The University of Edinburgh, Edinburgh, United Kingdom; 2 Institute for Biodiversity, Animal Health and Comparative Medicine, College of Medicine, Veterinary Medicine and Life Sciences, University of Glasgow, Glasgow, United Kingdom; 3 Tsetse and Trypanosomiasis Research Institute, Tanga, Tanzania; 4 Natural Resources Institute, University of Greenwich, Chatham Maritime, United Kingdom; National Institutes of Health, United States of America

## Abstract

**Background:**

Measuring the prevalence of transmissible *Trypanosoma brucei rhodesiense* in tsetse populations is essential for understanding transmission dynamics, assessing human disease risk and monitoring spatio-temporal trends and the impact of control interventions. Although an important epidemiological variable, identifying flies which carry transmissible infections is difficult, with challenges including low prevalence, presence of other trypanosome species in the same fly, and concurrent detection of immature non-transmissible infections. Diagnostic tests to measure the prevalence of *T. b. rhodesiense* in tsetse are applied and interpreted inconsistently, and discrepancies between studies suggest this value is not consistently estimated even to within an order of magnitude.

**Methodology/Principal Findings:**

Three approaches were used to estimate the prevalence of transmissible *Trypanosoma brucei s.l.* and *T. b. rhodesiense* in *Glossina swynnertoni* and *G. pallidipes* in Serengeti National Park, Tanzania: (i) dissection/microscopy; (ii) PCR on infected tsetse midguts; and (iii) inference from a mathematical model. Using dissection/microscopy the prevalence of transmissible *T. brucei* s.l. was 0% (95% CI 0–0.085) for *G. swynnertoni* and 0% (0–0.18) *G. pallidipes*; using PCR the prevalence of transmissible *T. b. rhodesiense* was 0.010% (0–0.054) and 0.0089% (0–0.059) respectively, and by model inference 0.0064% and 0.00085% respectively.

**Conclusions/Significance:**

The zero prevalence result by dissection/microscopy (likely really greater than zero given the results of other approaches) is not unusual by this technique, often ascribed to poor sensitivity. The application of additional techniques confirmed the very low prevalence of *T. brucei* suggesting the zero prevalence result was attributable to insufficient sample size (despite examination of 6000 tsetse). Given the prohibitively high sample sizes required to obtain meaningful results by dissection/microscopy, PCR-based approaches offer the current best option for assessing trypanosome prevalence in tsetse but inconsistencies in relating PCR results to transmissibility highlight the need for a consensus approach to generate meaningful and comparable data.

## Author Summary

Human African trypanosomiasis is a fatal disease that is carried by a tsetse vector. Assessing the proportion of tsetse which carries human-infective trypanosomes is important in assessing human disease risk and understanding disease transmission dynamics. However, identifying flies which carry transmissible infections is difficult, due to potential presence of other trypanosome species in the same fly, and concurrent detection of immature infections which are not transmissible. We used three methods to estimate the proportion of flies carrying human-infective trypanosomes: dissection and microscopic examination of flies to visualise trypanosomes directly in the fly; PCR of fly midguts in which trypanosomes were observed by microscopy; and theoretical analysis using a mathematical model of disease transmission. All three methods found the prevalence to be extremely low. Given the low prevalence, dissection/microscopy requires prohibitively large sample sizes and therefore PCR-based approaches are likely to be of most value. However, interpretation of PCR data is not straightforward; whilst PCR identifies flies carrying pathogen genetic material it does not directly identify flies with transmissible infections. This study highlights the need for a consensus approach on the analysis and interpretation of PCR data to generate reliable and comparable measures of the proportion of flies which carry transmissible human-infective trypanosomes.

## Introduction

For the vector-borne diseases, pathogen prevalence in a vector population is an indicator of disease risk, and accurate measures of the proportion of vectors carrying infections are needed for (i) guiding allocation of resources or targeting intervention programs [1]; (ii) monitoring the success of control interventions [2]; and (iii) as parameters in models of disease transmission which are increasingly used to predict disease distribution and persistence, and plan control interventions [3]. Approaches for detecting parasite prevalence in vector populations, known as xenomonitoring, have until recently usually relied on dissection of insect vectors and visualisation of parasites by microscopy, which is time consuming and reliant on operator skill. PCR has presented an alternative technique for several parasite-vector systems, e.g. *Plasmodium* spp [4], *Oncocerca volvulus* [5], [6], *Leishmania* spp. [7], [8], and the nematodes which cause lymphatic filariasis, *Wuchereria bancrofti*, *Brugia malaya* and *Brugia timori* [9], [10], generally having better ability to differentiate between species of similar morphology, increased sensitivity, and hence requiring smaller sample sizes [4], [6], [8].

Human African trypanosomiasis (HAT) is caused in East Africa by *Trypanosoma brucei rhodesiense* and transmitted by tsetse flies (*Glossina* spp). Measuring the prevalence of *T. b. rhodesiense* in the tsetse vector is of particular importance as HAT occurs in developing countries where resources for surveillance and disease control are limited [11] and knowledge of human disease risk is important for effective targeting of available resources. In addition, HAT is characterised by its focal nature, with human cases continuing over long periods of time in specific geographical areas, but the reasons for this persistence are not clear [12]. The prevalence of infection in tsetse is an important component in understanding transmission dynamics and detecting spatiotemporal trends, which have important implications for disease control.

Assessment of the prevalence of trypanosomes within tsetse populations has traditionally comprised dissection and microscopic examination of the mouthparts, midguts and salivary glands of the fly, relying on the differing development and maturation sites of the trypanosome subgenera to identify trypanosome species [13]. Trypanosomes found only in the mouthparts are classified as *Duttonella* or *vivax*-like, trypanosomes located in the mouthparts and midguts are classified as *Nannamonas* or *congolense*-type, and trypanosomes found in the midgut and salivary glands are *Trypanozoon* or *brucei*-like. When trypanosomes are found only in the midgut, the infection is assumed to be immature. This dissection/microscopy technique has several disadvantages for use in field studies: it is not possible to differentiate below the level of subgenus (for example *T. simiae* cannot be differentiated from *T. congolense*, since they share development sites in the fly); mature and immature infections cannot always be differentiated; and mixed infections cannot be identified or discriminated. Dissection and trypanosome identification are highly dependent on operator skill, and there exist variations in protocols, with some authors only examining the midgut and salivary glands if trypanosomes are found within the mouthparts [14], [15], whilst others examine all the organs [16], [17].

A suite of molecular tools has been developed for the trypanosomatids [18], [19]. PCR and sequence analysis techniques have served to overcome some of the disadvantages of dissection/microscopy and highlighted new information about tsetse-trypanosome interactions. PCR primers with high sensitivity and specificity now permit trypanosomes to be reliably identified to species or subspecies level, for example new strains or potentially even species of trypanosome have been identified [20], [21], [22], and human-infective *T. b. rhodesiense* and its morphologically-identical subspecies *Trypanosoma brucei brucei* (not pathogenic to man) can be accurately differentiated [23]. Mixed infections are common, with approximately one third of PCR positive flies carrying more than one trypanosome species [20], [24], [25] and up to four trypanosome species identified in individual flies [24], [25].

However, when it comes to assessing the prevalence of trypanosome infections in tsetse it is clear that the results generated by dissection/microscopy do not correlate well with data generated by PCR (for example only 38% [25] to 51% [24] of *Nannomonas* or *T. congolense*-like and *Duttonella* or *T. vivax*-like infections are classified as the same species by both techniques). For *T. brucei* sensu lato, with its potential for human infection, this presents a particular problem. In areas where *T. b. rhodesiense* is known to occur in wildlife and livestock hosts, and human cases are reported, the majority of studies of *T. brucei* s.l. in tsetse by dissection/microscopy show prevalence of zero, even when thousands of flies are examined [16], [26]. However when whole tsetse flies have been analysed by PCR surprising amounts of *T. brucei* s.l. DNA has been found, with 2% of *G. palpalis* and 18% of *G. pallidipes* testing positive [27], [28]. The discrepancy between dissection/microscopy and PCR highlights the issues of assessing the true prevalence of human infective trypanosomes in tsetse populations, particularly as it is not clear how these measures relate to transmissibility. Furthermore, it would be useful if a consensus could be reached as to how best to use molecular data, either alone or in combination with results of dissection/microscopy, to generate prevalence measures.

This study presents data from a persistent focus of Rhodesian HAT in the Serengeti National Park (SNP), Tanzania. Whilst cases of HAT have been reported in this area for over one hundred years [29], recent cases in both the local population and tourists have renewed public health concerns about the disease [30], [31]. With abundant populations of *G. swynnertoni* and *G. pallidipes*, and almost 100 000 tourists visiting the SNP each year in addition to resident staff and local populations [32], understanding and mitigation of human disease risk is a priority.

Previous studies carried out in SNP have relied on dissection/microscopy to determine tsetse prevalence (Table 1). Large scale studies in 1970 and 1971 failed to identify any salivary gland infections [16], [26] but a subsequent pooled rodent inoculation study detected nine out of 11000 *G. swynnertoni* flies (0.08%) infected with *T. brucei* s.l. [33]. These findings contrast with results of a more recent study that reported a prevalence of 3.0% for *T. brucei* s.l. in *G. swynnertoni* [34] and raise questions as to whether the wide variation in detected prevalence reflects real changes in tsetse infection levels and human exposure risk, or reflect methodological differences.

**Table 1:** Reported prevalence of *T. brucei* s.l. in the two main tsetse species in Serengeti National Park in previous studies.

	No. of flies examined	Prevalence*T. brucei* s.l.*(%)*	Technique	Reference
*G. swynnertoni*	6348	0	Dissection/microscopy	[16], [72]
	3550	0	Dissection/microscopy	[26]
	11040	0.08	Pooled rodent inoculation	[33]
	677	3.0	Dissection/microscopy	[34]
*G. pallidipes*	623	0	Dissection/microscopy	[16]
	199	0	Dissection/microscopy	[34]

This study assessed the prevalence of *T. brucei* s.l. and *T. b. rhodesiense* in the two main tsetse species in SNP, *G. swynnertoni* and *G. pallidipes*, using (i) dissection/microscopy and (ii) PCR analysis of infected midguts and salivary glands. A third approach was applied to infer the prevalence of *T. b. rhodesiense* in tsetse from a mathematical model of disease transmission, to examine whether previously reported low prevalences were consistent with other parameters that have been estimated for this system.

## Methods

### Tsetse Sampling

All field work was conducted in SNP, Tanzania, between October and November 2005 and August and October 2006. Tsetse sampling was carried out with the Tsetse and Trypanosomiasis Research Institute, Tanga, Tanzania. Seven sites were randomly selected for tsetse trapping in savannah and open woodland areas, within 1 km of roads and within a 40 km radius of park headquarters at Seronera, where tsetse dissection was conducted (coordinates UTM 36M (i) 711676, 9731432; (ii) 706816, 9733868; (iii) 710747, 9733536; (iv) 695691, 9727934; (v) 700825, 9746320; (vi) 693961, 9733122; (vii) 695278, 9741360). In each study site, three Epsilon traps [35] were installed for between five and eleven days, depending on trap catches. Each trap was situated at least 200 m from the next, and erected in mottled shade to reduce fly mortality. When placing traps, areas with fallen trees were avoided and traps were placed so that the entrances were directed towards gaps in vegetation, measures known to maximise fly catches by following the natural patterns of tsetse flight [36]. The location of each trap was recorded using a handheld global positioning system (Garmin Ltd, Kansas, USA). Traps were baited with 4-methylphenol (1 mg/h), 3-n-propylphenol (0.1 mg/), 1-octen-3-ol (0.5 mg/h) and acetone (100 mg/h) [37] and emptied twice daily.

### Dissection/Microscopy

All live non-teneral flies were dissected and labrum, hypopharynx, salivary glands and midgut examined for trypanosomes under 400× magnification [38]. For each fly, species, sex and the presence or absence of trypanosomes in each organ were recorded. To prevent contamination between flies and between different parts of each fly, dissection instruments were cleaned in 5% sodium hypochlorite, followed by rinsing in distilled water then phosphate buffered saline between each organ. Flies carrying trypanosome infections was categorised according to Lloyd and Johnson [13]. Confidence intervals were calculated using binomial exact 95% limits.

### Laboratory Analysis

All trypanosome-positive midguts and salivary glands were macerated in phosphate buffered saline and applied to FTA Classic cards (Whatman, Maidstone, UK) for further analysis. A subset of trypanosome-negative midguts was also preserved on FTA cards. FTA cards were allowed to dry for two hours and stored in foil envelopes with dessicant at ambient temperature prior to processing. For each sample, one disc of diameter 2 mm was cut out from the FTA card using a Harris Micro Punch™ tool. Between cutting of the sample discs, 10 punches were taken from clean FTA paper, to prevent contamination between samples. Discs were washed for two washes of 15 minutes each with FTA purification reagent (Whatman Biosciences, Cambridge, UK), followed by two washes of 15 minutes each with 1X TE buffer (Sigma Aldrich, Dorset, UK). Each disc was dried at room temperature for 90 minutes, and then used to seed a PCR reaction. After every seven sample discs, a negative disc was included and the punch tool and mat cleaned, to reduce the risk of contamination between discs, and ensure that any potential contamination would be detected. No evidence of contamination was seen in the sequence of dissection or PCR results.

TBR primers were used to detect a 177 bp satellite repeat sequence common to *T. b. brucei*, *T. b. rhodesiense* and *T. b. gambiense* [39]. PCR was carried out in 25 µl reaction volumes containing 16.0 mM (NH4)2SO4, 67 mM Tris-HCl, 0.01% Tween 20 (NH4 buffer, Bioline Ltd, London, UK) 1.5 mM MgCl2, 800 µM total dNTP's, 0.4 µM of each primer TBR1 and TBR2, 0.7 Units of BioTaq Red DNA polymerase (Bioline Ltd, London, UK) and one washed disc. For samples testing positive for *T. brucei* s.l., *T. b. rhodesiense* was differentiated from *T. b. brucei* by detection of the serum-resistance associated (SRA) gene. Simultaneous amplification of another single copy gene, a phospholipase C (PLC) sequence found in *T. brucei* s.l., confirmed that there was sufficient *T. brucei* s.l. material present in the sample to detect the presence of *T. b. rhodesiense* [40]. SRA PLC PCR was carried out in duplicate in a 25 µl reaction volume containing 3 mM MgCl, 1.25 µl of Rediload dye (Invitrogen, Karlsbad, California), 1.5 Units Hot StarTaq (Qiagen, Crawley, UK), 0.2 µM of each primer and one washed disc. The SRA gives a 669 bp product, with a PLC band at 324 bp.

For all PCRs, one negative control (water) and one positive control (genomic DNA) were run for every 16 samples, in addition to negative control blank discs. PCR products were run on a 1.5% (w/v) agarose gel at 100 V, stained with ethidium bromide and visualised under an ultraviolet transilluminator.

### Calculations of prevalence

Detection of *T. b. rhodesiense* in a tsetse midgut does not indicate a mature infection as only a small proportion of midgut infections will develop to mature infections in the salivary glands. The following calculation was used to predict the prevalence of mature transmissible *T. b. rhodesiense* infections, where *Dis_pos_* is the proportion of flies with midguts which were positive by dissection/microscopy, *PCR_pos_* is the proportion of these which tested positive by PCR, *P_Tbr/Tbb_* is the proportion of *T. brucei* s.l. positive flies with sufficient genetic material present (ie give positive results with PLC PCR) which test positive for *T. b. rhodesiense* (as determined by SRA PCR) and *P_mat_* is the proportion of immature *T. b. rhodesiense* infections which develop to maturity in the salivary glands, estimated to be 0.12 (CI 0.10–0.14), [41], [42]:
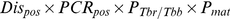
(1)This calculation relies on three assumptions: (i) that dissection/microscopy is 100% sensitive for detecting trypanosome infections in tsetse midguts, and that all flies carrying *T. brucei* s.l. will have midgut infectons; (ii) that TBR PCR has 100% sensitivity and specificity for detection of *T. brucei* s.l. in tsetse midguts; (iii) that SRA PCR has 100% sensitivity and specificity for detection of *T. b. rhodesiense*, if the sample is positive on PLC PCR. The implications of potential assumption violations on the prevalence estimate are addressed in the discussion.

Confidence intervals were calculated by repeat sampling from nested distributions of the data. Since the value for *P_mat_* was taken from Milligan et al. (1995) the distribution of the original data was used, where *Y* is the number of flies with midgut infections and *P_mat_* is the proportion of these which developed mature salivary gland infections (*Y* = 1133, *P_mat_* = 0.12). Potential values were generated by sampling from the following nested distributions with 10 000 iterations, and ninety five percent confidence intervals calculated by taking the 2.5% and 97.5% quantiles of the values obtained: *n_1_∼binom(N, Dis_pos_), n_2_∼binom(n_1_, PCR_pos_), n_3_∼binom(n_2_, P_Tbr/Tbb_), p_1_∼binom(Y, P_mat_), n_4_∼binom(n_3_, p_1_/1133)*.

### Models

Rogers' [43] model of vector-borne trypanosome transmission was adapted for one host population (wildlife, *x*) and two vector populations (*G. swynnertoni*, *y_1_* and *G. pallidipes*, *y_2_*). Although occasional cases of human African trypanosomiasis do occur, the rate of human feeding by tsetse is very low [0.1% of feeds on blood meal analysis, 16], so the human population was not included in the model. The model is described by the following equations:
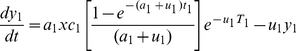
(2)

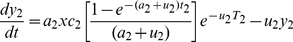
(3)

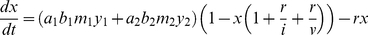
(4)that were simultaneously solved using the lsoda function in the package odesolve in R (http://www.r-project.org/) to give equilibrium conditions for the prevalence of *T. b. rhodesiense* in wildlife hosts, *G. swynnertoni* and *G. pallidipes* and which could be compared to empirically derived estimates of prevalence.

Parameters were based on those described by Rogers [43] but adjusted to reflect infection in wildlife (Table 2). Parameters specific to *T. b. rhodesiense*, and to *G. swynnertoni* and *G. pallidipes*, were used where possible. The proportion of tsetse developing salivary gland infection after feeding on an infected cow is 16% for *G. morsitans* (closely related to *G. swynnertoni*) and 2.1% for *G. pallidipes* [44]; however wildlife exhibit a degree of trypanotolerance and generally show low parasitaemia [45], which reduces the probability that a feeding tsetse will develop infection, also indicated by very low infection rates in tsetse fed on wildlife experimentally [46], [47]. A number of wildlife species do not appear to develop infection with *T. brucei* s.l., either proving uninfectible in experimental infections eg baboons [46] or rarely observed with natural infection despite being popular hosts for tsetse, eg elephant [16], [48], [49], so the probability that an infected tsetse feeding on a host results in an infection is also lower compared to cattle. The incubation period of 18 days follows that of Dale *et al.* [50] for laboratory infections of *T. b. rhodesiense* in *G. morsitans* flies; no specific data were available for *G. pallidipes* so the same value was used. Wildlife host parameters have been chosen to represent all wildlife species. Duration of incubation period and duration of infection are therefore estimated mean values from experimental infections of wildlife [46], [51], [52]. Although age prevalence patterns suggest the development of some immunity to *T. brucei* s.l. in lions [53], experimental infections do not indicate a clear immune period in other species [46]. SNP has high densities of both wildlife [54] and tsetse [34].

**Table 2:** Parameters for one-host, two-vector population model of *Trypanosoma brucei rhodesiense* transmission.

Parameter		Value		Reference
Host parameters				
Duration of infection in wildlife hosts	*1/r*	30 days		[46], [52]
Incubation period in wildlife hosts	*1/i*	7 days		[46], [51]
Duration of immunity in wildlife hosts	*1/v*	1 day		[46], [52]
Tsetse parameters		*G.swynnertoni*	*G.pallidipes*	
Ratio of vectors to wildlife hosts (density flies per km/density hosts per km)	*m*	10000/40	5000/40	[54], [75]
Proportion of infected fly bites producing infection in wildlife hosts	*b*	0.15	0.15	[43], [46]
Feeding rate on wildlife (proportion meals from wildlife/duration feeding cycle in days)	*a*	100/3	100/3	[16], [26], [76]
Fly mortality	*u*	0.03	0.03	[43]
Incubation period in tsetse	*T*	18	18	[50]
Proportion of meals from infected hosts which develop into mature infections in tsetse	*c*	0.016	0.0021	[44], [46], [47]
Age below which tsetse susceptible to infection	*t*	1	1	[43]

All statistical analyses and model solving were carried out using R 2.12.1 (The R Foundation for Statistical Computing, http://www.r-project.org).

## Results

### Dissection/microscopy

In total, 6455 tsetse were dissected and examined, comprising 4356 *G. swynnertoni* (2759 females, 1597 males) and 2099 *G. pallidipes* (1472 females, 627 males). Overall, trypanosomes were observed (in mouthparts, midgut, or both) in 9.2% of *G. swynnertoni* (females 10.2%, males 7.5%), and 3.7% of *G. pallidipes* (females 3.9%, males 3.2%) examined. No salivary gland infections were observed. Using the classical trypanosome species identification based on the location of parasites within the fly, the prevalence of *T. vivax*-like, *T. congolense*-like and *T. brucei*-like trypanosomes is shown in Table 3.

**Table 3:** Prevalence of trypanosomes in tsetse by dissection and microscopy.

	*G. swynnertoni*	*G. pallidipes*
Mouthpart only*T. vivax* group	6.43(5.7–7.2)	2.20(1.6–2.9)
Mouthpart/midgut*T. congolense* group	2.11(1.7–2.6)	1.24(0.80–1.8)
Salivary gland*T. brucei* group	0(0–0.085)	0(0–0.18)

Trypanosomes were identified by dissection and microscopic examination of tsetse and classified according to the criteria of Lloyd and Johnson [13]. Confidence limits are 95% exact binomial confidence intervals.

### PCR Analysis

For 5428 flies (all those sampled in 2006), all midguts where trypanosomes were observed (n = 133) were analysed by PCR (Table 4). No flies were found with salivary gland infections. The prevalence of flies with trypanosomes in the midgut on dissection/microscopy, which were also midgut PCR positive (*Dis_pos_*×*PCR_pos_*, assumed to represent *T. brucei s.l.* immature infections) was 0.83% in *G. swynnertoni* and 0.71% in *G. pallidipes*. All midguts that tested positive for *T. brucei* s.l. were further analysed with SRA PCR, with 10 out of 43 PLC positive and 1 of these SRA positive, therefore the proportion of *T. brucei* s.l. testing positive for *T. b. rhodesiense* was 0.1. Using the expression in Eq. 1, this gives a predicted prevalence of transmissible *T. b. rhodesiense* infections of 0.010% for *G. swynnertoni* and 0.0085% for *G. pallidipes* (Table 4). The prevalence was also calculated separately by sex and using sex-specific maturation ratios of 0.21 for males and 0.044 for females [41]. The predicted prevalence of *T. b. rhodesiense* mature infections in *G. swynnertoni* was 0.016% for males (the number of flies testing positive on dissection/microscopy and PCR out of the total number examined was 11/1448) and 0.0035% for females (20/2289), and in *G. pallidipes* was 0.019% for males (5/541) and 0.0024% for females (7/1151).

**Table 4:** Prevalence of *T. b. rhodesiense* in tsetse through incorporation of dissection/microscopy and PCR data.

Tsetse species	*Number of tsetse examined*	*Number positive dissection/microscopy*	Number positive by PCR for *T. brucei* s.l.	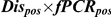	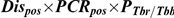	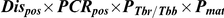
*G. swynnertoni*	3737	104	31	0.83%	0.083%	0.010%CI 0–0.054
*G. pallidipes*	1691	29	12	0.71%	0.071%	0.0085%CI 0–0.059

All fly midguts where trypanosomes were observed by microscopy were analysed by PCR., *Dis_pos_* is the proportion of flies examined that were positive by dissection/microscopy, *PCR_pos_* is the proportion of dissection/microscopy positive flies that were also positive by PCR for *T. brucei* s.l., *P_Tbr/Tbb_* is the ratio of *T. b. rhodesiense* to *T. b. brucei* and *P_mat_* is the proportion assumed to mature to the salivary glands. CI are 95% confidence intervals.

Midguts from 78 flies with no trypanosomes observed on microscopy were also analysed by PCR. Of these, 3.8% (n = 3) tested positive for *T. brucei* s.l.. None of these tested positive with PLC or SRA.

### Model

Assuming equilibrium, the model yielded prevalences of *T. b. rhodesiense* of 0.0064% in *G. swynnertoni* and 0.00085% for *G. pallidipes*. The model predicted the prevalence of *T. b. rhodesiense* in wildlife hosts to be 2.5%, which is within the range of reported prevalences in wildlife in SNP of 1.8% and 4.3% [55], [56].

The results of all three approaches are presented in Table 5.

**Table 5:** Prevalence of *T. b. rhodesiense* by dissection/microscopy, PCR and model inference.

	Prevalence of *T. b. rhodesiense* (%)		
	Dissection/microscopy	PCR	Model
*G. swynnertoni*	0 (0–0.085)	0.010 (0–0.054)	0.0064
*G. pallidipes*	0 (0–0.18)	0.0089 (0–0.059)	0.00085

Prevalence of *T.b.rhodesiense* in the two main tsetse species in Serengeti National Park was analysed by dissection/microscopy and model inference. Dissection/microscopy cannot differentiate *T. brucei brucei* and *T. b. rhodesiense* so is a measure of *T. brucei* s.l. prevalence. Ninety-five percent confidence limits are shown in parentheses.

## Discussion

In this study we present data obtained from three different approaches to measuring the prevalence of transmissible *T. b. rhodesiense* infections in tsetse populations in Serengeti National Park. Fundamental difficulties have been identified associated with the detection of trypanosome infections in tsetse, requiring new approaches to move beyond generation of infection prevalence data to make inferences about transmissibility. The three approaches used in this study confirmed the prevalence of *T. b. rhodesiense* in SNP to be very low. The prevalence of *T. brucei* s.l. measured by dissection/microscopy was zero, despite confirmation by the other techniques that *T. brucei* s.l. was circulating in the area, and evidence of infection in wildlife and human hosts, highlighting a common problem with this technique. The results from PCR analysis of tsetse midguts were used to generate a measure of transmissible infections. In addition, a mathematical model of disease transmission used to predict the prevalence of transmissible infections based on other parameters for this system, confirmed the low prevalence gained by other approaches was compatible with the prevalence of *T. b. rhodesiense* in wildlife hosts reported in SNP. This study highlights specific challenges in measuring transmissible *T. b. rhodesiense* infections in tsetse, which have important implications for assessing this variable, and interpreting temporal and spatial patterns of infection in affected areas of Africa.

These results illustrate the difficulties of dissection/microscopy techniques, which in this study estimated the prevalence of *T. brucei* s.l. in tsetse populations as zero, despite strong evidence to indicate the presence of infection in tsetse using other techniques, and evidence for circulation of *T. b. rhodesiense* in vertebrate hosts in the same area [30], [31], [55]. The low prevalence commonly obtained through dissection/microscopy is often attributed to low diagnostic sensitivity of this technique, and there is evidence that some infections which would be classed as immature by microscopy may actually be transmissible. For example, inoculation of trypanosomes found in the mouthparts from flies with trypanosomes present in the mouthparts and midgut by dissection did give rise to *T. brucei* s.l. infections in mice, both in laboratory and field studies [57], [58], and PCR of dissection-negative salivary glands revealed additional *T. brucei* s.l. infected flies in *Glossina palpalis palpalis* in Cote d'Ivoire [59]. Whilst this may play a part in the low prevalence observed, the use of other techniques in this study confirmed the prevalence to be extremely low, and the prevalence of zero by dissection/microscopy in this study is more likely attributed to insufficient sample size than low sensitivity. With a prevalence of 0.01% (the highest of the estimates in this study) it would be necessary to examine around 30 000 flies to detect a difference from zero with 95% confidence.

Dissection/microscopy has a number of other disadvantages: it is time consuming and requires skilled technicians, and whilst it does not require substantial investment in technology, this may be outweighed by high staff costs. Identification of species, mixed infections and immature infections is unreliable, particularly if other trypanosome species are also of interest. Furthermore dissection/microscopy alone cannot differentiate between *T. b. brucei* and *T. b. rhodesiense*. The dissection/microscopy technique was first discussed in detail by Lloyd and Johnson in 1924 as an alternative to cumbersome rodent inoculation studies. However, Lloyd and Johnson relied principally on morphology of the developmental and infective forms, using the location within the fly only as an additional aid. It is clear that in areas where the prevalence is very low, dissection is less than ideal. However, since the majority of historical studies have relied on dissection/microscopy it is important to understand how these data compare to those generated by other techniques if we want to be able to detect temporal trends.

PCR-based techniques have the potential to provide a sensitive and specific tool to identify flies carrying *T. b. rhodesiense*. We found that 30% of microscopy-positive midguts tested positive for *T. brucei* s.l. by PCR in *G. swynnertoni* and 41% in *G. pallidipes*. It is difficult to compare these directly with other studies as protocols vary widely, but between 7.9% and 19% of microscopy-positive midguts have been reported testing positive for *T. brucei* s.l. in these tsetse species [20], [21], [25]. However, a PCR positive fly does not indicate a transmissible infection, but only indicates the presence of trypanosomal DNA. Here we have combined PCR data with information on the proportion of immature *T. b. rhodesiense* infections which mature to the salivary glands to estimate the prevalence of mature transmissible infections. The prevalence was within the confidence limits of dissection/microscopy and similar to the predictions of the model. Prevalence was higher in males than females, reflecting the increased probability of maturation in males [41]. Although in this study, dissection/microscopy were carried out prior to PCR, the increased likelihood of detecting immature *T. brucei* s.l. in midguts by PCR means the sample size can be lower for the equivalent precision, reducing field costs and time compared to the substantial sample sizes needed for dissection/microscopy only.

The calculation used to predict the prevalence of mature *T. b. rhodesiense* infections by incorporating dissection/microscopy and PCR data relied on assumptions regarding the sensitivity of dissection/microscopy for detecting midgut trypanosome infections, and the diagnostic sensitivity and specificity of TBR and SRA PCRs when used on tsetse midgut samples. Whilst identification of trypanosomes in the midgut is widely used in the laboratory there is little data available on the sensitivity of this technique in the field. There is however no evidence to suggest that flies can carry *T. brucei* s.l. without trypanosomes being present in the midgut. TBR and SRA PCRs have high specificity [40], [60]. Whilst the analytical sensitivity of TBR and SRA PCRs is known (they are both able to detect 0.1 pg of trypanosome genetic material or less, equivalent to one trypanosome [39], [40]), there is no quantitative data on the diagnostic sensitivity when used on tsetse samples. The diagnostic sensitivity of TBR on blood samples from livestock is 76% [60]; however the number of parasites in tsetse midgut samples is several fold higher than the parasitaemia in livestock (which is often <10 trypanosomes/ml [40]) hence diagnostic sensitivity is likely to be considerably higher for tsetse samples.

Imperfect test sensitivity and specificity can significantly affect prevalence estimates, particularly when the prevalence is very low [61]. Ideally the sensitivity and specificity of each technique would have been included in the analysis to produce prevalence estimates and confidence intervals that reflect this information. The paucity of data to examine these assumptions illustrates the importance of more critical assessment of these techniques, but likely reflects the difficulty of assessing sensitivity and specificity in the absence of a gold standard technique. In the absence of quantitative data, the most likely violation of the assumptions is that the sensitivity of each technique is not 100% hence the prevalence may have been underestimated.

In this study, 10% *T. brucei* s.l. infections were identified as *T. b. rhodesiense*. Whilst this is not outside the range of values found in previous studies [62], a proportion of one third has been more commonly reported [63]. SRA PCR targets a single copy gene, and therefore requires the presence of a large amount of parasite DNA. Despite an initial sample size of over 6000 flies, only ten infected midguts had sufficient genetic material present to check for *T. b. rhodesiense*, so our estimate of the proportion of *T. brucei* s.l. which are *T. b. rhodesiense* is not very precise (10%, CI 0.2–44%). Using the value of 33% resulted in a prevalence of *T. b. rhodesiense* in *G. swynnertoni* of 0.03% and in *G. pallidipes* of 0.028%.

It is interesting that 3.8% of microscopy-negative flies tested positive for *T. brucei* s.l. by PCR. Previous authors have found high prevalences of *T. brucei* s.l. by PCR (for example 18% [27]), and there are potential explanations for this high detection rate. Flies that test positive on PCR but were microscopy-negative may result from the presence of trypanosomal DNA (known to be detectable for over 10 days in the absence of live trypanosomes [64]) or a very small number of trypanosomes for example in a recent blood meal where trypanosomes are not able to establish an infection. Experimentally it has been established that only around 12–43% of susceptible flies feeding on an infected host will develop an immature infection even in teneral flies [44], [65]. In older flies, the majority of trypanosomes ingested will not develop further. Simple calculations illustrate that if trypanosomal DNA is detectable for 10 days, flies feed every 3 days and 5% of hosts carry *T. brucei* s.l., at any one time, up to 17% of flies may have detectable *T. brucei* s.l. DNA, in the absence of an immature or mature infection.

Given the drawbacks of using other techniques, it is reassuring that a model incorporating independently estimated parameters for this system predicted similar values for the prevalence of *T. b. rhodesiense* in tsetse. Whilst it might seem questionable whether the very low prevalence found by the other techniques is consistent with the reported prevalence of *T. b. rhodesiense* in wildlife hosts of 1.8–4.3% [55], [56], a simple equilibrium-based model analysis showed that with *T. b. rhodesiense* prevalence in wildlife of 2.5%, the prevalence in tsetse remains below 0.01%, and consistent with field measures. For diseases such as HAT where low prevalence raises diagnostic challenges, broad agreement of prevalence estimates using quite different approaches permits a measure of confidence in each.

A constraint to going forwards with making assessments of prevalence is the absence of a gold standard technique for identifying transmissible *T. b. rhodesiense* infections in tsetse. Dissection/microscopy requires prohibitive samples sizes and potentially may not detect all transmissible infections; PCR techniques based on amplification of DNA from midguts rely on assumptions of factors which are known to vary and tests for which the diagnostic performance is poorly defined; models require accurate knowledge of all other parameters in a system and assumptions regarding equilibrium dynamics. Even rodent inoculation may miss infections as rodents often fail to become infected due to their innate resistance to infection. However, approaches for the future are likely to rely on PCR based techniques so it is important that reliable and comparable protocols are developed. Currently, there are many different approaches reported for using PCR data to look at *T. brucei* s.l. in tsetse populations, including PCR of any organs found infected [25] (similar to this study although we did not include mouthparts), PCR of all organs in the fly if any organ is found infected on dissection/microscopy [59], [66] and PCR of whole tsetse flies [for example 27], [28]. This variety of protocols raises two important issues:

To interpret data from PCR analysis it is important to be clear what PCR results do or do not represent. For example, identification of *T. brucei* s.l. DNA by PCR in whole flies does not indicate a mature and therefore transmissible infection, but only the prevalence of *T. brucei* s.l. DNA. Is it possible to use this measure as a direct indicator of risk? This approach has been taken for other pathogens. For example in assessing prevalence of West Nile virus in mosquitoes, most screening programs test the whole mosquito, detecting mosquitoes with any trace of WNV present, rather than testing the salivary glands, which would give the rate of transmissible infections [1]. PCR studies to identify the nematodes which cause lymphatic filariasis in mosquito populations give a prevalence of infected mosquitoes, but cannot differentiate between pre-infective L1 and L2 larvae, and infective L3 larvae [10]. However this approach is more common where detecting pathogen presence or absence is the main aim, so the exact nature of the relationship between presence of pathogen DNA and transmissible infections is less critical. Approaches measuring the prevalence of *T. brucei* s.l. or *T. b. rhodesiense* DNA, either in infected midguts, in all midguts or in whole flies, are assuming a constant relationship between this measure, and the prevalence of transmissible infections (in turn assumed to represent human risk). In this study we relied on experimental measures of the proportion of midgut *T. b. rhodesiense* infections which mature to the salivary glands to estimate the prevalence of transmissible infections. However there are two areas for concern with this assumption: (i) laboratory studies may not accurately reflect the situation in the field; and (ii) this proportion is known to vary with factors such as sex, levels of certain antioxidants, mating in female flies, and environmental factors such as temperature [41], [67]. While this approach may be suitable for obtaining an approximate measure of prevalence, the validity of the assumptions would be challenged by comparative studies over different spatial or temporal situations where these factors are likely to vary. Interpretation of PCR results from analysis of whole flies or from midguts without prior dissection/microscopy is more problematic. This study illustrates the high proportion of microscopy-negative midguts which test positive by PCR and similar findings are reported from PCR of whole flies [27], [28]. It is not known how the proportion of flies testing positive by this technique relates to the prevalence of transmissible infections. Approaches involving PCR of salivary glands may hold most promise. PCR of microscopy-negative salivary glands or salivary drops has been shown to increase the prevalence compared to dissection/microscopy alone both in the field [59] and the laboratory [68]. It is not clear what these discrepancies between microscopic and PCR analysis of salivary glands means with regard to transmission and this is an area where further research is required.

The second concern is with respect to comparative data analysis, in that the variety of techniques used means it is difficult to assess trends in prevalence. This is a significant problem – prevalences measured in different ways cannot be compared between different areas or times, making it impossible to detect changing disease dynamics and human disease risk, and hindering our understanding of the complex relationships between trypanosomes, hosts and vectors. Agreement on an optimal protocol for the collection and interpretation of data on trypanosome prevalence in tsetse populations would be helpful in generating more comparable data.

This study shows that the prevalence of *T. b. rhodesiense* in *G. swynnertoni* and *G. pallidipes* in SNP can be sustained at very low levels. Both the PCR data and the model suggest that *G. pallidipes* may play a role, albeit a lesser one, in *T. b. rhodesiense* transmission as well as *G. swynnertoni*, which has always been regarded as the important vector species in Serengeti. The two species differ in both feeding preferences and vector competence; while both species include suids and bovids in their diet, *G. swynnertoni* feeds predominantly on warthog while *G. pallidipes* feeds predominantly on buffalo [69], [70]. Although both *G. swynnertoni* and *G. pallidipes* are known to avoid feeding on man, this effect is particularly evident for *G. pallidipes* [71], which likely decreases the importance of this species in human disease transmission. The prevalence found in this study is consistent with that of previous studies by dissection/microscopy [16], [26], [72] so we did not find any evidence of long term trends in disease transmission. However, the prevalence in this study does differ significantly from that reported in 2007 of 3% [34]. Whilst this may reflect temporal or spatial variation in prevalence within SNP, our model suggests that a sustained prevalence this high is very unlikely.

The low prevalence of *T. b. rhodesiense* in tsetse found in this study suggests that the risk of HAT to tourists is low. Odour-baited tsetse traps are known to target older flies [73]; flies which bite people are usually younger and less likely to be carrying a transmissible infection since the prevalence of mature infections increases with age [74]. This is consistent with the low number of cases (<5 per year) reported in Serengeti, in comparison to the large number of visitors (almost 100,000 per year [32]). However, the risk of encountering an infected fly is higher in those who spend extended periods exposed to tsetse in SNP, so it should be ensured that adequate screening and treatment provision is in place to detect cases in park and lodge staff.

In conclusion **t**he prevalence of transmissible human infective trypanosomes in tsetse populations is an important parameter but there is no ideal diagnostic test to measure it. While new molecular diagnostic tools offer great potential for epidemiological studies, many challenges remain in the interpretation of field data generated from these tools, and these need to be recognised and addressed. Development of protocols that directly measure the prevalence of transmissible infections, and the consistent application of such protocols, would aid our knowledge of human disease risk, allow detection of spatial and temporal trends in disease transmission and add to our understanding of complex disease systems.
